# Characteristics of Asymptomatic Bacteriuria in Diabetes Mellitus Patients: A Retrospective Observational Study

**DOI:** 10.7759/cureus.13562

**Published:** 2021-02-26

**Authors:** Muhammad Sohaib Asghar, Mohammed Akram, Manjeet Singh, Farah Yasmin, Rabail Yaseen, Nisar Ahmed, Mariam Siddiqui, Maira Hassan, Uzma Rasheed, Abraish Ali

**Affiliations:** 1 Internal Medicine, Dow University of Health Sciences, Karachi, PAK; 2 Internal Medicine, Liaquat National Hospital, Karachi, PAK; 3 Internal Medicine, Dow International Medical College, Karachi, PAK

**Keywords:** multi-drug resistant bacteria, urine culture, cultural sensitivity, antimicrobial resistance, antibiotic susceptibility, minimum inhibitory concentration (mic), urinary tract infection, pan-drug resistance, diabetes type 2, diabetic nephropathy (dn)

## Abstract

Background and objective

The term asymptomatic bacteriuria (ASB) refers to the isolation of bacteria in a urine specimen of individuals without any symptoms of urinary tract infection (UTI). Diabetes mellitus (DM) is a disease involving multiple organ systems, characterized by its chronicity and hence endless complications including ASB. This study aimed to determine the characteristics of ASB and antibiotic susceptibility patterns among patients with diabetes.

Materials and methods

This was a retrospective observational study conducted in a tertiary care hospital. The study included patients with a diagnosis of diabetes with no signs and symptoms of UTI but who still showed the growth of an organism in urine culture. A total of 222 urine cultures were analyzed retrospectively, ensuring that they met the inclusion criteria through non-probability consecutive sampling.

Results

The mean age of the study participants was 62.89 ± 13.77 years; 76% of them were females, and 61% had a family history of diabetes. The most frequent organisms isolated were Escherichia coli (E. coli), Enterococcus, Klebsiella pneumonia, Pseudomonas aeruginosa, and Enterobacter species. A total of 20 subjects had dual bacterial growth in their cultures, with Enterococcus species (n=17) being the most common organism. Gender, family history of diabetes, levels of hemoglobin A1c (HbA1c), and advanced age were all found significantly associated with ASB.

Conclusion

Our study is the first of its kind to analyze and examine the risk factors associated with ASB in DM patients, and to identify the pathogens involved, along with assessing their antibiotic resistance profiles.

## Introduction

Diabetes mellitus (DM) is a disease involving multiple organ systems. Its hallmark is its chronicity, and hence it is associated with endless complications that occur due to a defect in the production or utilization of insulin, a hormone that helps to regulate blood sugar levels by transporting glucose to different tissues for uptake when the glucose levels in the blood reach above a standard level of concentration [1-3]. According to the International Diabetes Federation's data from 2019, approximately 463 million adults worldwide were found to have this disease, out of which 79% belonged to countries with low socioeconomic status [4]. According to a survey conducted in Pakistan in 2017, the prevalence of type 2 DM is reported to be 16.98% in the country [4]. With the prevalence of this disease on the rise and expected to increase further, DM has become a hot topic of discussion in the field of medicine, ultimately leading to further elaboration of disease processes that can ensue after its initial manifestation. DM is notorious for causing cardiovascular, neurological, and renal insults [3]. DM has a negative impact on humoral immunity and neutrophilic function; it also damages the antioxidant system, evidently making patients prone to infections of any organ system of the body, including urinary tract infection (UTI) [1,5-6]. Even after treatment, re-infection is common among diabetic patients [1,7-8]. In light of this, asymptomatic bacteriuria (ASB) among diabetics poses an imminent threat of progressing from urethritis to life-threatening pyelonephritis in no time.

ASB is defined as the existence of bacteria in the urine of a patient who has not shown any symptoms or signs of a UTI [8]. It is detected via urine culture of a properly collected urine specimen as per certified medical guidelines [8]. While Escherichia coli (E. coli) is the most common organism isolated from urine specimens of both diabetic and non-diabetic patients, atypical pathogens along with increased antimicrobial resistance to all pathogens are found more in the urine cultures of the former. Due to an increased risk of upper urinary tract involvement and the likelihood of ASB progressing to a symptomatic UTI in this patient population, it is recommended to have urine samples cultured both pre- and post-treatment [9].

Diabetic patients with ASB often tend to experience pyelonephritis and albuminuria [10]. Previous studies have shown that women with diabetes are three times more likely to have ASB than those without diabetes [11]. Both diabetic men and women are more likely to be admitted for a complication of ASB, acute pyelonephritis, than those without the disease. This condition affects 3.4-17% of diabetic males as compared to non-diabetic males [12]. In contrast, this condition affects diabetic women 6-24 times more than non-diabetic women [12]. Aside from the increased risk of acquiring pyelonephritis, diabetic patients face an increased risk of having to deal with the complications of it as well [13], the most serious of which, and more likely to occur among this patient population, being renal and peri-nephric abscess, emphysematous pyelonephritis, renal papillary necrosis, bacteremia, and urosepsis [1,12]. Furthermore, it has been proposed in previous studies that diabetic patients with ASB may deteriorate with greater frequency toward renal failure [12]. Although treatment for ASB until it causes complications have not yet been indicated in the diabetic population, cultured and identified gram-positive or gram-negative bacteria are found to be susceptible to the treatment with the drugs gentamicin and ciprofloxacin respectively, among many others [11,14].

Once diagnosed with DM, patients require lifelong insulin therapy or adjunctive therapy with this hormone to control glycemic levels [3]. Adequate metabolic control not only limits complications of the disease but also lowers the risk of acquiring infection in an already susceptible diabetic patient. In addition to adequate management, WHO recommends monitoring for adverse effects that can occur due to this disease [13]. Among the interventions suggested is screening for pathologies that can later proceed to manifest as kidney diseases [7]. In view of these guidelines and the projected rise of DM, especially in countries with low socioeconomic status, and considering the paucity of data regarding the findings of ASB among the diabetic population in Pakistan, we conducted this study with the primary objective of determining the characteristics of ASB in DM patients.

## Materials and methods

This was a retrospective observational study conducted at the internal medicine department of a tertiary care hospital. The medical records of all patients were accessed after obtaining ethical approval from the institutional review board. A sample size of 218 was calculated by using the Raosoft sample size calculator (http://www.raosoft.com/samplesize.html), from a total population size of 500, response distribution (anticipated frequency of outcome factor in the population) of 50%, confidence interval of 95%, and margin of error of 0.05. ASB is diagnosed either based on the presence of 100,000 colony-forming units (CFU)/ml in a midstream clean catch urine sample or on the presence of 100 CFU/ml in the urine sample obtained from a catheterized patient [8]. According to the Clinical and Laboratory Standards Institute (CLSI) guidelines, clean voided, midstream urine samples were collected from all the subjects and processed following standard guidelines. Urine gram stain examination was performed to look for pus cells and bacteria. In patients with significant bacteriuria, antibiotic susceptibility was performed. For diagnosing ASB in females, two consecutive specimens with the isolation of the same species in quantitative counts of at least 100,000 CFUs/ml of urine were considered. Whereas in males, a single specimen with one bacterial species isolated in a quantitative count of at least 100,000 CFUs/ml was considered.

A proforma was designed with two sections; the first section covered demographic details including name (optional), age (in years), gender, family history of DM, and comorbidities including hypertension, chronic kidney disease (CKD), and others, while the second section was further divided into two sub-sections. The first sub-section included the space to document signs and symptoms of UTI (any patient with features of UTI was not included in the study). The second sub-section included the level of hemoglobin A1c (HbA1c), gram staining, and a list of organisms causing UTIs and antibiotics that are usually used to treat it. The documented urine cultures of the study population having positive bacterial growth were included. All those patients who were diagnosed with DM and were not having any signs and symptoms of UTI at the time of admission but had a positive urinary culture were included in our study. All those patients who did not have DM, those who had signs and symptoms of UTI, or those having negative urinary culture were excluded from the study. The study also excluded all symptomatic UTI patients, pregnant females, and all those who used antimicrobial medications in the last two weeks prior to the study.

After the data was collected through the non-probability consecutive sampling method. It was analyzed on IBM SPSS Statistics version 25.0 (IBM, Armonk, NY) and results were obtained. The statistical difference was calculated via independent sample t-test, chi-square test, and Fisher’s exact test. A p-value of <0.05 was considered statistically significant.

## Results

Out of the 222 included subjects, there were three times as many women as men, and they had a slightly younger mean age of 61.54 ± 13.86 years as compared to men (p=0.007). Two-thirds of the study subjects with a positive urinary culture were in the age group of 50-75 years. Around 58% of males had a family history of DM, in contrast with 62% of females; 28.37% of females had a known history of CKD in contrast with 9% among males; 36% of females had hypertension while only 14.28% among males had it. Ischemic heart disease was found in 4.95% of females and 4.50% of males. Almost 40% of the subjects had HbA1c in the range of 6.5-9.0%, 22% had it in the range of 5.7-6.5%, and the remaining percentage segments of the subjects had it equally distributed in the ranges of <5.7%, 9-11.5%, 11.6-14.0%, and >14.0% respectively (Table 1).

**Table 1:** Demographic data of the study population (n=222) *Indicates independent sample t-test; **indicates Fisher’s exact test; ***indicates chi-square test M: males; F: females; DM: diabetes mellitus; IHD: ischemic heart disease; CVA: cerebrovascular accident; BPH: benign prostatic hyperplasia; HIV: human immunodeficiency virus; CKD: chronic kidney disease; TB: tuberculosis; HbA1c: hemoglobin A1c

Demographic data							P-value
1	Age group distribution among patients, years	Age group	Frequency		Percentage		0.059**
		<25	3		1.4		
		25-50	36		16.2		
		50-75	148		66.7		
		>75	35		15.8		
		Total	222		100.0		
2	Mean age (in years)	62.89 ± 13.77					0.007*
		Males: 67.20 ± 12.67; females: 61.54 ± 13.86					
3	Gender	Males: 23.9% (n=53); females: 76.1% (n=169)					0.580***
4	Family history of diabetes	Present: 61.7% (n=137); absent: 38.3% (n=85)					
5	Comorbidities (alongside DM)	None other than DM: 43.24% (n=96)		M: 14.41% (n=32)		F: 28.82% (n=64)	0.006**
		Hypertension: 39.63% (n=88)		M: 11.26% (n=25)		F: 28.37% (n=63)	
		IHD: 9.45% (n=21)		M: 4.50% (n=10)		F: 4.95% (n=11)	
		CVA: 13.96% (n=31)		M: 7.20% (n=16)		F: 6.75% (n=15)	
		Autoimmune: 1.35% (n=3)		M: 0.0% (n=0)		F: 1.35% (n=3)	
		BPH: 9.90% (n=22)		M: 9.90% (n=22)		F: 0.0% (n=0)	
		HIV: 0.09% (n=2)		M: 0.04% (n=1)		F: 0.04% (n=1)	
		CKD: 37.38% (n=83)		M: 9.00% (n=20)		F: 28.37% (n=63)	
		TB: 1.35% (n=3)		M: 0.09% (n=2)		F: 0.04% (n=1)	
6	Levels of HbA1c among the diabetic population (n=222)	Level of HbA1c (%)	Frequency		Percentage		0.341**
		<5.7	16		7.2		
		5.7-6.5	49		22.1		
		6.5-9.0	89		40.1		
		9.0-11.5	37		16.7		
		11.5-14	17		7.7		
		>14	14		6.3		
		Total	222		100.0		
7	Mean HbA1c (%)	8.46 ± 2.64					0.784*
		Males: 8.54 ± 2.76; females: 8.43 ± 2.60					
8	Number of cultures included in the study (n=222)	No. of Isolated single micro-organisms: 90.99% (n=202)					0.789**
		Dual growth with both bacteria: 9.00% (n=20)					

E. coli, Enterococcus species, and Klebsiella pneumonia were the most common organisms isolated from the urine samples of the subjects, with the frequency of 54%, 13%, 11% respectively among females, and 49%, 11%, and 9% respectively among males (Table 2).

**Table 2:** Frequency of isolated micro-organisms in the urine cultures among genders (n=222) ^1^Most prevailing organism among both genders; ^2^second most prevailing organism among both genders *Chi-square test​​​​​​; ^**^Fisher’s exact test E. coli: Escherichia coli; MRSA: methicillin-resistant Staphylococcal aureus

Organisms	Males (n=53)	Females (n=169)	P-value
Staphylococcus aureus (n=4)	01 (2%)	03 (2%)	1.000**
Acinetobacter species (n=2)	01 (2%)	01 (0.6%)	0.421**
Klebsiella oxytoca (n=3)	01 (2%)	02 (1%)	0.561**
Burkholderia cepacia (n=1)	00 (0%)	01 (0.6%)	1.000**
Enterobacter species (n=8)	02 (4%)	06 (3.5%)	1.000**
Citrobacter species (n=1)	01 (2%)	00 (0%)	0.239**
MRSA (n=2)	02 (4%)	00 (0%)	0.056**
Enterococcus species^2 ^(n=28)	06 (11%)	22 (13%)	0.745*
Streptococcus species (n=3)	00 (0%)	03 (2%)	1.000**
Pseudomonas aeruginosa (n=8)	03 (6%)	05 (3%)	0.400**
E. coli^1 ^(n=118)	26 (49%)	92 (54%)	0.493*
Klebsiella pneumonia (n=23)	05 (9%)	18 (11%)	0.800*
Proteus vulgaris (n=0)	00 (0%)	00 (0%)	-
Proteus mirabilis (n=1)	01 (2%)	00 (0%)	0.239**
Dual growth (of any two bacteria in a single culture) (n=20)	04 (8%)	16 (9%)	0.789**

Dual growth was observed in 20 subjects, out of which 16 were females; 43.75% of the dual growth among females isolated E. coli and Enterococcus species while 18.75% isolated Enterococcus with Klebsiella species. On the contrary, 75% of the dual growth among males showed the growth of Enterococcus and E. coli while 25% showed Enterococcus and Proteus mirabilis (Table 3).

**Table 3:** Frequency of micro-organisms among genders in dual growth cultures (n=20) Fisher’s exact test was used to compute all the p-values *Most common dual growth among both genders; **second most common dual growth among males; ***second most common dual growth among females E. coli: Escherichia coli; MRSA: methicillin-resistant Staphylococcal aureus

Dual growth organisms (combinations)	Males (n=4)	Females (n=16)	P-value
E. coli and Enterococcus species*	03 (75%)	07 (43.75%)	-
Enterococcus and Klebsiella pneumonia***	00 (0%)	03 (18.75%)	
Enterococcus and MRSA species	00 (0%)	01 (6.25%)	
Klebsiella pneumonia and Pseudomonas aeruginosa	00 (0%)	01 (6.25%)	
E. coli and Streptococcus species	00 (0%)	01 (6.25%)	
Pseudomonas aeruginosa and Streptococcus species	00 (0%)	01 (6.25%)	
Enterococcus and Enterobacter species	00 (0%)	01 (6.25%)	
Enterococcus and Proteus mirabilis**	01 (25%)	00 (0%)	
Enterococcus and Klebsiella oxytoca	00 (0%)	01 (6.25%)	
Frequency of isolated dual growth organisms	-		
E. coli (n=11)	3 (75%)	8 (50%)	0.591
Enterococcus species (n=17)	4 (100%)	13 (81%)	1.000
Klebsiella pneumonia (n=4)	0 (0%)	4 (100%)	0.538
Streptococcus species (n=2)	0 (0%)	2 (100%)	1.000
Pseudomonas aeruginosa (n=2)	0 (0%)	2 (100%)	1.000
MRSA (n=1)	0 (0%)	1 (100%)	1.000
Proteus mirabilis (n=1)	1 (25%)	0 (0%)	0.200
Klebsiella oxytoca (n=1)	0 (0%)	1 (100%)	1.000
Enterobacter species (n=1)	0 (0%)	1 (100%)	1.000

Of all the subjects, 62% had a positive family history of DM, out of which 64% were diagnosed with E. coli infection as opposed to 36% without a family history (p=0.009). Patients diagnosed with other organisms did not have a family history of diabetes. Out of the 20 subjects who had a dual growth in their cultures, 13 had a positive family history of DM (Table 4).

**Table 4:** The association between frequency of micro-organisms (both single/dual growth cultures combined) and family history of diabetes mellitus (n=222) ^1^Most common bacteria in patients with a positive family history; ^2^second most common bacteria in patients with positive family history *P-value calculated by chi-square test; **p-value calculated by Fisher’s exact test E. coli: Escherichia coli; MRSA: methicillin-resistant Staphylococcal aureus

Organisms	Family history present (n=137)	Family history absent (n=85)	P-value
Staphylococcus aureus (n=4)	01 (0.7%)	03 (3.5%)	0.158**
Acinetobacter species (n=2)	00 (0%)	02 (2.3%)	0.146**
Klebsiella oxytoca (n=4)	02 (1.4%)	02 (2.3%)	0.638**
Burkholderia cepacia (n=1)	00 (0%)	01 (1.2%)	0.383**
Enterobacter species (n=9)	08 (5.8%)	01 (1.2%)	0.158**
Citrobacter species (n=1)	00 (0%)	01 (1.2%)	0.383**
MRSA (n=3)	02 (1.4%)	01 (1.2%)	1.000**
Enterococcus species^2^ (n=45)	24 (17.5%)	21 (24.7%)	0.195*
Streptococcus species (n=5)	03 (2.1%)	02 (2.3%)	1.000**
Pseudomonas aeruginosa (n=10)	05 (3.6%)	05 (5.8%)	0.512**
E. coli^1^ (n=129)	89 (64.9%)	40 (47.0%)	0.009*
Klebsiella pneumonia (n=27)	15 (10.9%)	12 (14.1%)	0.483*
Proteus vulgaris (n=0)	00 (0%)	00 (0%)	-
Proteus mirabilis (n=2)	01 (0.7%)	01 (1.2%)	1.000**
Dual growth (of any two bacteria) (n=20)	13 (9.4%)	07 (8.2%)	0.751*

Among the cultures that returned positive, E. coli (n=129) was found most sensitive to amikacin (84.49%) followed by fosfomycin (80.62%) and gentamicin (66.67%). It was found resistant to ampicillin (86.04%) followed by cefixime (82.94%) and cefuroxime (82.17%). Among the 27 positive cultures of Klebsiella pneumonia, the most sensitive antibiotic was fosfomycin (70.37%) followed by amikacin (55.56%) and piperacillin/tazobactam (44.44%); they were found to be resistant to ceftriaxone (77.78%), cefixime (77.78%), and cefuroxime (74.07%) respectively. Pseudomonas was positive in 10 cultures; it was most sensitive to ciprofloxacin (70%) followed by ceftazidime (60%) and amikacin (60%), while being most resistant to ceftazidime (40%), as shown in Table 5.

**Table 5:** Frequency of susceptibility of micro-organisms to antibiotics isolated in the cultures (n=222) *Most sensitive/resistant antibiotic among the particular organism; ^second most sensitive/resistant antibiotic among the particular organism Staph aureus: Staphylococcal aureus; MRSA: methicillin-resistant Staphylococcal aureus; P. aeruginosa: Pseudomonas aeruginosa; E. coli: Escherichia coli; K. pneumonia: Klebsiella pneumonia; K. oxytoca: Klebsiella oxytoca

Antibiotics		Organisms													
		1. Staph aureus	2. MRSA	3. Enterococcus	4. Streptococcus	5. P. aeruginosa		6. E. coli	7. K. pneumonia	8. Proteus mirabilis	9. Acinetobacter	10. K.oxytoca	11. Burkholderia cepacia	12. Enterobacter	13. Citrobacter
Aztreonam	Sensitive	0	0	0	0	0		0	0	0	0	0	0	0	0
	Resistant	0	0	0	0	1		1	2	0	0	0	0	0	0
Amikacin	Sensitive	4*	2^^^	2	0	6^^^		109*	15^^^	1^^^	0	2*	0	3	1*
	Resistant	0	1^^^	1	0	3^^^		8	10	0	1^^^	2^^^	0	5^^^	0
Amoxicillin/clavulanic acid	Sensitive	1	0	21^^^	5*	0		57	8	2*	0	2*	0	0	0
	Resistant	0	3*	1	0	0		58	16	0	0	2^^^	0	0	0
Ampicillin	Sensitive	0	0	20	5*	0		4	1	2*	0	0	0	0	0
	Resistant	0	0	20^^^	0	0		111*	2	0	0	1	0	0	0
Ciprofloxacin	Sensitive	3^^^	0	1	0	7*		33	4	1^^^	0	1^^^	0	2	0
	Resistant	1*	1^^^	5	0	3^^^		87	19	0	2*	3*	0	7*	0
Ceftriaxone	Sensitive	0	0	1	3	0		20	5	1^^^	1^^^	1^^^	0	2	0
	Resistant	0	0	3	0	0		99	21*	0	1^^^	2^^^	0	7*	1*
Cefixime	Sensitive	0	0	2	0	0		22	5	1^^^	0	1^^^	0	2	0
	Resistant	0	0	1	0	0		107^^^	21*	0	0	3*	0	7*	1*
Cefuroxime	Sensitive	0	0	1	0	0		22	6	1^^^	0	1^^^	0	2	0
	Resistant	0	0	2	0	0		106	20^^^	0	0	3*	0	7*	1*
Co-trimoxazole	Sensitive	0	0	2	1	0		36	3	1^^^	0	1^^^	1*	2	0
	Resistant	1*	0	1	1	0		57	14	0	2*	2^^^	0	1	1*
Gentamicin	Sensitive	4*	1	3	0	4		86	11	0	0	1^^^	0	4^^^	0
	Resistant	0	1^^^	1	0	3^^^		33	14	0	1^^^	3*	0	5^^^	1*
Meropenem	Sensitive	0	0	1	0	4		85	11	0	2*	0	1*	3	1*
	Resistant	0	0	0	0	2		7	7	0	0	2^^^	0	4	0
Piperacillin/tazobactam	Sensitive	0	0	3	0	7*		81	12	1^^^	2*	1	0	2	1*
	Resistant	0	0	0	0	3^^^		34	13	0	0	2^^^	0	5^^^	0
Vancomycin	Sensitive	3^^^	3*	18	1	0		0	0	0	0	0	0	0	0
	Resistant	0	0	12	0	0		0	1	0	0	0	0	0	0
Fosfomycin	Sensitive	0	0	32*	4^^^	1		104^^^	19*	2*	1^^^	1^^^	0	4^^^	1*
	Resistant	0	0	7	1	0		16	5	0	0	1	0	4	0
Nitrofurantoin	Sensitive	3^^^	1	13	1	0		51	8	0	0	1^^^	0	0	1*
	Resistant	0	0	5	0	0		2	1	0	0	1	0	1	0
Levofloxacin	Sensitive	0	0	7	3	0		4	1	1^^^	0	0	0	0	1*
	Resistant	0	0	26*	2*	1		8	5	0	0	1	1*	3	0
Colomycin/polymyxin B	Sensitive	0	0	0	0	2		6	8	0	0	2*	0	5*	0
	Resistant	0	0	0	0	0		0	0	0	0	0	0	0	0
Tobramycin	Sensitive	0	0	0	0	0		0	0	0	0	0	0	0	0
	Resistant	0	0	0	0	1		6	7	0	0	2^^^	0	4	0
Sulbactam/cefoperazone	Sensitive	0	0	0	0	0		0	0	0	0	0	0	0	0
	Resistant	0	0	0	0	1		6	8	0	0	2^^^	0	4	0
Ceftazidime	Sensitive	0	0	0	0	6^^^		0	0	0	0	0	1*	0	0
	Resistant	0	0	0	0	4*		0	0	0	0	0	0	0	0
Linezolid	Sensitive	0	0	15	0	0		0	0	0	0	0	0	0	0
	Resistant	0	0	0	0	0		0	0	0	0	0	0	0	0
Cloxacillin	Sensitive	4*	0	0	0	0		0	0	0	0	0	0	0	0
	Resistant	0	3*	0	0	0		0	0	0	0	0	0	0	0
Organisms							Minimal inhibitory concentration								
1. Methicillin-sensitive Staphylococcal aureus (n=4)							Ampicillin: susceptibility ≥29 mm, resistant ≤28 mm; amoxicillin + clavulanic acid: susceptibility ≥22 mm, resistant ≤21 mm; cefuroxime: susceptibility ≥24 mm, resistant ≤20 mm; ciprofloxacin: susceptibility ≥21 mm, resistant ≤15 mm; co-trimoxazole: susceptibility ≥16 mm, resistant ≤10 mm; gentamicin: susceptibility ≥15 mm, resistant ≤12 mm								
2. MRSA (n=2)															
3. Enterococcus species (n=24)							Ampicillin: susceptibility ≥17 mm, resistant ≤16 mm; amoxicillin + clavulanic acid: susceptibility ≥17 mm, resistant ≤16 mm; ciprofloxacin: susceptibility ≥21 mm, resistant ≤15 mm								
4. Streptococcus species (n=4)							Ampicillin: susceptibility ≥24 mm, resistant ≤19 mm; ciprofloxacin: susceptibility ≥17 mm, resistant ≤13 mm								
5. Pseudomonas aeruginosa (n=8)							Amikacin: susceptibility ≥17 mm, resistant ≤14 mm; aztreonam: susceptibility ≥22 mm, resistant ≤15 mm; ciprofloxacin: susceptibility ≥21 mm, resistant ≤15 mm; meropenem: susceptibility ≥19 mm, resistant ≤15 mm; gentamicin: susceptibility ≥15 mm, resistant ≤12 mm; piperacillin/tazobactam: susceptibility ≥21 mm, resistant ≤14 mm								
6. E. coli (n=82)							Amikacin: susceptibility ≥17 mm, resistant ≤14 mm; aztreonam: susceptibility ≥21 mm, resistant ≤17 mm; ciprofloxacin: susceptibility ≥21 mm, resistant ≤15 mm; meropenem: susceptibility ≥23 mm, resistant ≤19 mm; gentamicin: susceptibility ≥15 mm, resistant ≤12 mm; piperacillin-tazobactam: susceptibility ≥21 mm, resistant ≤17 mm								
7. Klebsiella pneumonia (n=20)							Amikacin: susceptibility ≥17 mm, resistant ≤14 mm; aztreonam: susceptibility ≥21 mm, resistant ≤17 mm; ciprofloxacin: susceptibility ≥21 mm, resistant ≤15 mm; meropenem: susceptibility ≥23 mm, resistant ≤19 mm; gentamicin: susceptibility ≥15 mm, resistant ≤12 mm; piperacillin-tazobactam: susceptibility ≥21 mm, resistant ≤17 mm								
8. Proteus mirabilis (n=1)							Amikacin: susceptibility ≥17 mm, resistant ≤14 mm; aztreonam: susceptibility ≥21 mm, resistant ≤17 mm; cefuroxime: susceptibility ≥18 mm, resistant ≤14 mm; ciprofloxacin: susceptibility ≥21 mm, resistant ≤15 mm; meropenem: susceptibility ≥23 mm, resistant ≤19 mm; gentamicin: susceptibility ≥15 mm, resistant ≤12 mm								
9. Acinetobacter species (n=1)							Amikacin: susceptibility ≥17 mm, resistant ≤14 mm; aztreonam: susceptibility ≥21 mm, resistant ≤17 mm; ciprofloxacin: susceptibility ≥21 mm, resistant ≤15 mm; meropenem: susceptibility ≥18 mm, resistant ≤14 mm; gentamicin: susceptibility ≥15 mm, resistant ≤12 mm; piperacillin-tazobactam: susceptibility ≥21 mm, resistant ≤17 mm								
10. Klebsiella oxytoca (n=4)							Not checked								
11. Burkholderia cepacia (n=1)															
12. Enterobacter species (n=9)															
13. Citrobacter species (n=1)															

Among the patients with CKD (n=83), Enterococcus was the most likely organism to be positive in urine culture, with 57.8% being infected (n=26), while the rest (42.2%, n=19) infecting non-CKD patients (p=0.002). While E. coli was positive in only 35 CKD patients (27.1%), the rest of 94 cultures (62.9%) were found in non-CKD patients (p=0.0002). Enterobacter was exclusively present in the cultures of patients with CKD, with all the nine growths (100%), as opposed to no growth in non-CKD patients (p=0.0001). With respect to patients presenting with no other comorbidities except for DM (n=96), E. coli was the most likely associated organism (n=52) with a p-value of 0.001, while Enterococcus was more likely to occur in patients with multiple comorbidities (p=0.010). With respect to patients with benign prostatic hyperplasia (BPH) (n=22), E. coli was found infecting 50% of their cultures (n=11), among others (p=0.561).

## Discussion

Many studies have investigated the risk factors associated with ASB in DM patients; however, few such studies have been conducted in Pakistan. It is important to identify the risk factors associated with ASB in diabetic patients, considering how the prevalence of DM is increasing in the Pakistani population and how ASB in patients with their associated comorbidities can lead to adverse outcomes. This study aimed to outline the associated risk factors of ASB in DM, the pathogens involved, and their antibiotic sensitivity profiles. The risk factors for ASB include proteinuria/albuminuria, raised creatinine levels, female gender, elevated HbA1c, longer duration of diabetes, prior UTI history, overuse of antibiotics, and use of sodium-glucose cotransporter-2 (SGLT2) inhibitors.

Various studies have shown demographic factors (particularly age and female gender) to be associated with ASB in DM patients. Our study validates those findings with results showing that the mean age of our patients was high (62.89 ± 13.77 years), which was similar to the mean ages reported by Turan et al. (60.8 ± 9.5 years), Geerlings et al. (63.0 ± 10 years), and Odetoyin et al. (65.52 ± 9.4 years) [12,15-16]. The mean age in our study, however, differed from the findings of Hamdan et al., Boroumand et al., and Zhanel et al., all of whom reported lower mean ages [17-19]. The highest percentage of patients with ASB in our study were found in the 50-75 years age range, with 66.7% of the total study population falling in that group. This age range was comparable to that reported by Meiland et al, where 61.8% of the diabetic patients positive for ASB were in the age range of 56-75 years [20].

Another significant demographic factor that should be considered is the female gender; 76.1% of our study population were women. Various studies have confirmed the role of the female sex as a risk factor for ASB in diabetics, and our results fell along these lines. These results are strikingly similar to those reported by Turan et al., where 77.2% of females were positive for ASB (although in this study the female sex was not a significant factor) [12]. Studies by Jha et al. (70%) and Alebiosu et al. (72.7%) reported similar results as well, while Banerjee et al. reported a lower frequency (59%) [21-23]. Bissong et al. and Matteucci et al., however, recorded higher percentages of women in their studies at 86.4% and 86% respectively and concluded that the female gender was a positive risk factor for ASB [14,24]. The high prevalence of women in our study population can be due to the fact that most of the women had undergone postmenopausal changes (the mean age of women in this study was 61.54 ± 13.86 years). This would result in the deficiency of female sex hormones and would cause atrophic changes in the vagina and alter the vaginal flora, thus resulting in a higher percentage of women with ASB [25]. Even though BPH is a highly prevalent comorbidity in elderly males, the frequency of ASB in BPH was quite low as compared to other comorbidities in our study population (9.90%). It can be postulated that even though BPH is considered a significant risk factor of ASB, it is more likely to cause symptomatic urinary infections rather than asymptomatic ones.

The role of glycemic control (HbA1c) as a risk factor for diabetic patients to develop ASB has been controversial. Studies in the literature are both for and against its role as a risk factor. Our results show a significant association between the HbA1c levels and the risk of diabetic patients developing ASB. Around 89 (40.1%) of our subjects had an HbA1c between 6.5-9.0%. The overall mean HbA1c of the subjects was 8.46 ± 2.64%. Turan et al. have confirmed in their study that poor glycemic control is a risk factor for ASB [12]. The mean HbA1c in their study was similar to ours (8.7 ± 2.0%). A study in India among diabetic patients also revealed a positive relation between HbA1c and ASB; however, 71.43% of their patients had an HbA1c of >7% [26]. Matteucci et al. concluded that HbA1c was indeed a risk factor for the development of ASB, but their mean HbA1c was 7.9 ± 1.1%, which was slightly lower compared to our results [24]. Bonadio et al. also confirmed a positive correlation between HbA1c and ASB, but their mean HbA1c of 9.6 ± 2.0% was higher than our findings [9]. Geerlings et al. did report a similar mean HbA1c among their study population (8.5 ± 1.6%) but concluded that poor glycemic control was not a risk factor for ASB [15]. The mean HbA1c reported by Zhanel et al. (13.3 ± 4.1%) was higher than our results, but they too concluded that HbA1c and ASB are not related [19]. Similar conclusions have been reached by Nicolle et al. and He et al. [11,27]. The inconsistent results regarding the relationship between glycemic control and ASB reflect the different study populations and selection criteria used in these studies.

The most common comorbidity (besides DM, which was present in all the subjects) in our study was CKD, which was present in 37.38% of our population. Zhanel et al. reported that 5.9% of their patients had kidney disease, which is much lower than our findings [19]. The high prevalence of CKD in our study could be due to diabetic nephropathy, or the collective effect of ASB and DM on the kidneys. A study showed that patients with type 1 DM and ASB did experience an increase in their creatinine clearance [4]. An interesting result in our study was related to the pathogens cultured in patients with CKD as comorbidity. Our results showed that Enterococcus was present in 57.8% of these patients. The second most common comorbidity in our study was hypertension, which was present in 53 patients (23.87%). This is higher than the 12% prevalence reported by Meiland et al. [20]. Papazafiropoulou et al. reported 79.5% of their patients to be hypertensive, while even higher frequencies were found by Kasyan et al. (87.3%) [25,28].

Our study found three organisms to be the most common among patients with ASB, namely E. coli (53.3%), Enterococcus (18.6%), and Klebsiella pneumonia (11.15%). Multiple studies have reported a variety of pathogens in their results. A study that is very much in line with our findings was conducted in Sudan, which also found E. coli (56.4%), Klebsiella pneumonia (23.0%), and Enterococcus faecalis (12.8%) to be the most common organisms [17]. A study conducted in Southern India among diabetic patients had similar organisms cultured from their patients, but with dissimilar frequencies [22,29]. Klebsiella pneumonia (42.4%), E. coli (21.2%), and Enterococcus faecalis (12.1%) were isolated in the study in Nigeria [22], while E. coli (67%), Enterococcus (9%), and Klebsiella (14%) were found in the study conducted in Southern India [29]. Zhanel et al. found a prevalence of E. coli (52.9%) that is similar to ours, but also isolated Streptococcus species (11.4%) and Staphylococcus species (5.9%) in their study [19]. Odetoyin et al. performed a study where the isolated organisms were Staphylococcal aureus (80.9%), Klebsiella (9.5%), Enterococcus faecalis (4.8%), and E. coli (4.8%) [16].

We found that E. coli was mainly susceptible to aminoglycosides and fosfomycin, with resistance shown predominantly to ampicillin and third-generation cephalosporins. Nigussie et al. found that E. coli was sensitive to nitrofurantoin (100%), norfloxacin (90.9%), ciprofloxacin (81.8%), but resistant to ampicillin (100%) and trimethoprim-sulfamethoxazole (81.8%) [30]. This study also showed that E. coli was resistant to gentamicin (72.7%), but our study showed it was susceptible to gentamicin (66.7%). The resistance of E. coli in this study to ampicillin was 100%, while our results showed the resistance to be 86.04%. Our study showed a 67.4% resistance to ciprofloxacin as compared to the 81.8% sensitivity in the study by Nigussie et al. [30]. Bissong et al. found that E. coli was resistant to nalidixic acid (33.3%), gentamicin (26.7%), and cefuroxime (13.3%) [14]. These results differed from ours as gentamicin was sensitive in 66.7% of the organisms, and the resistance of cefuroxime was much higher in our study (82.17% vs. 13.3%). A study conducted in India found E. coli to be 100% sensitive to imipenem, but 90% resistant to third-generation cephalosporins, thus resembling the resistance pattern of E. coli shown in our study [26].

Klebsiella pneumonia was susceptible to fosfomycin (70.37%) followed by amikacin (55.56%) and piperacillin/tazobactam (44.44%) while showing resistance mostly to cephalosporins and lower resistance to amoxicillin. A study in China showed that Klebsiella pneumonia was resistant to amoxicillin (100%), cephalothin (50%), and cefuroxime (41.7%) [27]. Alebiosu et al. found that Klebsiella pneumonia was susceptible to gentamicin and ciprofloxacin in 85.7% of the organisms, nitrofurantoin in 78.5%, and ofloxacin in 71.4% [22]. Our antibiotic profile for Klebsiella pneumonia showed its susceptibility to gentamicin in 40.7% cases, to ciprofloxacin in 14.8%, and to nitrofurantoin in 29.6%, thus showing that the susceptibility of the organism to all antibiotics was much lower.

The most sensitive antibiotics for Enterococcus were fosfomycin (71.1%), amoxicillin (46.67%), and vancomycin (40%). Enterococcus showed resistance mainly to levofloxacin (57.78%) and ampicillin (44.44%). He et al. showed that Enterococcus in their studies was resistant to levofloxacin as well (33.3%), but the resistance to the antibiotic seen in our study was greater [27]. Hamdan et al. documented ampicillin to be sensitive to 80% of the organisms, while we found the sensitivity to ampicillin in our study was much lower [17]. A study in Southern India placed the sensitivity of the pathogen to vancomycin at 89%, which was much greater when compared to the sensitivity to the same antibiotic in our study [30]. 

This study has a few limitations. The study was conducted at a single tertiary care hospital in an urban location. Moreover, the aspects of sexual hygiene, socioeconomic status, urinary catheterization, other risk factors for ASB, and the duration of diabetes were not taken into account in our analysis.

## Conclusions

Despite many studies present in the literature about ASB in DM patients, with diverse protocols and study populations, data regarding the risk factors of ASB in DM patients in Pakistan is scarce. In this regard, our study is the first of its kind to analyze and study the risk factors associated with ASB in DM patients, and to identify the pathogens involved, along with assessing their antibiotic resistance profiles. Based on our results, we recommend that more similar studies be conducted in the region, to improve and develop guidelines and protocols on managing patients with ASB and DM. Based on the limited data that we obtained from our study, we would recommend that doctors or nephrologists plan to start antibiotics prophylactically with caution because of a high prevalence of resistance to antibiotics. Hence, we recommend that antibiotics be used prophylactically only if the patient has two or more comorbidities, in order to prevent the complications that can be caused by ASB, such as pyelonephritis, perinephric abscess, and renal scarring.
